# The Creation of an Average 3D Model of the Human Cartilaginous Nasal Septum and Its Biomimetic Applications

**DOI:** 10.3390/biomimetics8070530

**Published:** 2023-11-06

**Authors:** Peter S. Han, Nihal Punjabi, Mickey Goese, Jared C. Inman

**Affiliations:** 1 Department of Otolaryngology–Head and Neck Surgery, Loma Linda University Medical Center, Loma Linda, CA 92350, USA; 2 Department of Head and Neck Surgery, University of California Los Angeles, Los Angeles, CA 90095, USA; 3 Case Western Reserve University School of Medicine, Cleveland, OH 44106, USA; 4 Independent Researcher

**Keywords:** nasal septum, septoplasty, anatomic shape, 3D printing, finite elemental analysis

## Abstract

The cartilaginous nasal septum is integral to the overall structure of the nose. Developing our an-atomic understanding of the septum will improve the planning and techniques of septal surgeries. While the basic dimensions of the septum have previously been described, the average shape in the sagittal plane has yet to be established. Furthermore, determining the average shape allows for the creation of a mean three-dimensional (3D) septum model. To better understand the average septal shape, we dissected septums from 40 fresh human cadavers. Thickness was measured across pre-defined points on each specimen. Image processing in Photoshop was used to superimpose lateral photographs of the septums to determine the average shape. The average shape was then combined with thickness data to develop a 3D model. This model may be utilized in finite elemental analyses, creating theoretical results about septal properties that are more translatable to real-world clinical practice. Our 3D septum also has numerous applications for 3D printing. Realistic models can be created for educational or surgical planning purposes. In the future, our model could also serve as the basis for 3D-printed scaffolds to aid in tissue regeneration to reconstruct septal defects. The model can be viewed at the NIH 3D model repository (3DPX ID: 020598, Title: *3D Nasal Septum*).

## 1. Introduction

The nasal septum is a bony cartilaginous structure that serves as a major buttress in the overall structure of the nose. It is composed of the quadrangular cartilage, also known as the cartilaginous septum, the perpendicular plate of the ethmoid bone, and the vomer bone. Deviations in the nasal septum, often secondary to trauma, can affect symptomatic quality of life, thus requiring temporary measures to alleviate symptoms or definitive surgical repair.

A septoplasty is a surgical procedure for addressing a deviated septum. Modern septoplasty involves removing deviated portions of cartilage and bone while preserving the surrounding perichondral flap. An adequate strut of cartilage—the L-strut—is also preserved to support the external nose and avoid sequelae such as tip ptosis or a Pollybeak deformity. When most of the septum is severely deviated, an extracorporeal septoplasty may be undertaken to remove, reconstruct, and reinsert the entire septum. Depending on the location and severity of the septal deviation, a septoplasty may be performed concurrently with a rhinoplasty, which addresses the external framework of the nose and often uses grafts harvested from the septum [1].

Several studies have been conducted on the anatomy of the quadrangular cartilage because the manipulation of this structure significantly impacts the aesthetic and functional outcomes following septal surgery [2,3,4]. In addition to the gross dimensional measurements of length, width, and thickness, shape characterizes three-dimensional (3D) structures and has not been thoroughly evaluated in the cartilaginous septum literature. Septal shape is often only addressed in the context of septal deviation pathology, which focuses on the coronal plane. However, to the best of our knowledge, the overall shape of the healthy cartilaginous septum in the sagittal plane has not been reported [5]. Given that the contour and angulation of the L-strut is largely dependent on the native septal shape, expanded knowledge of the septal shape would improve our biomechanical understanding of the L-strut. Previous studies that have examined L-strut strength are often based on just one individual septum. This may limit the generalizability of these studies, as the septum is known to have significant variability in its shape [6]. Thus, characterizing the average septum shape not only improves our anatomical understanding of this structure, but also makes investigations into its properties more widely applicable to a broad patient population.

Elucidating septal shape also allows for more advanced septal modeling. By combining shape with measurements of thickness, a 3D septal model can be created, which has several implications for 3D printing (3DP). Three-dimensional printing is a form of additive manufacturing, where a material is gradually layered to develop the final product. This technology allows for the precise and fast production of custom physical objects [7]. While 3DP’s original application was in manufacturing, a wide variety of biocompatible materials are now available that enable the control of the macro- and microstructure of physical models, implants, and prostheses [8]. Thus, 3DP has found a natural home in medicine and has been increasingly used across several fields, including otolaryngology, maxillofacial surgery, orthopedics, neurosurgery, and cardiac surgery [9]. While the initial investment in the technology and technical skills required for 3DP is high, the cost is likely offset by decreased surgical times and decreased reliance on outside manufacturers [7].

The objective of this study is to determine the average shape of the cartilaginous septum from multiple individual specimens. Then, we aim to create a 3D model of the septum based on this shape. Extrapolating the average shape from several individual septums has never been conducted before, to the best of our knowledge. Doing so ensures our 3D mean septal model is anatomically accurate and representative of the diversity in septal shape. Consequentially, this model will allow for increased generalizability in future investigations on septal properties. An average 3D septal model also provides an opportunity to advance 3DP in the fields of rhinology and facial plastic surgery, ultimately improving medical education and clinical practice.

## 2. Materials and Methods

### 2.1. Dissection

The cartilaginous septum was sharply dissected from 40 non-embalmed, fresh human cadaver heads which were less than 48 h post-mortem. The mean and standard deviation (SD) of age at death was 80.4 ± 12.8 years (range = 50–89). Of these, 20 were male and 20 were female. This study was exempt from review by our institutional review board, as no living patients were included. The scientific and ethical protocols of the Loma Linda University School of Medicine Anatomy Department were followed.

**Figure 1:**
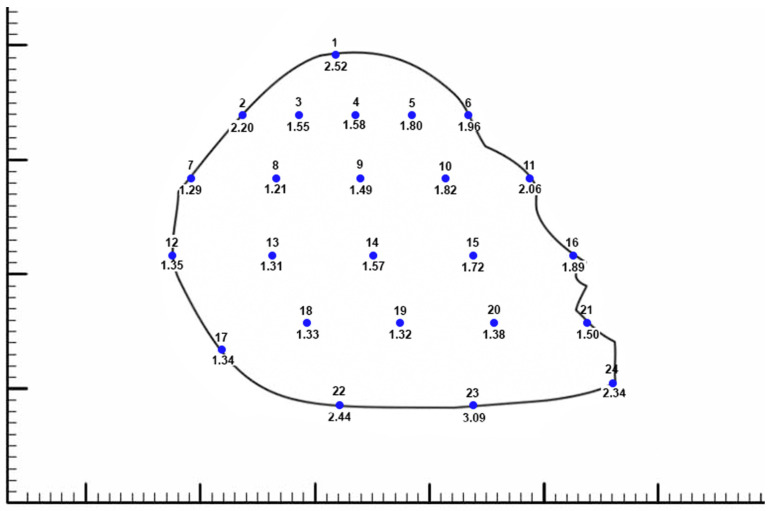
Average thickness measured at 24 predefined points from all 40 cartilaginous septums. The numbers below each point represent the average thickness at that point, measured in millimeters. The small hash marks on the scale represent 1 mm. The septum shape silhouette presented represents the 50th percentile shape that we developed into a 3D model.

A small border of bone was carefully removed with the cartilaginous septum to prevent the fracture or warping of the cartilage when the specimen was manipulated and removed from the nasal cavity. The thickness of each dissected cartilaginous septum was then measured with 0.01 mm resolution digital calipers in a grid-like format. Thickness measurements were taken at intervals of 5 mm across the horizontal length of the septum, starting from both fixed and relative points along the anterior curvature of the septum. The established fixed points were the keystone area, the anterior septal point, and the posterior caudal septal point. The relative points created were the midpoint between the anterior septal point and the posterior caudal septal point along the caudal anterior border of the septum, as well as two equidistant points set between the anterior septal point and the keystone point along the anterior dorsal septum. This allowed for thickness measurements to be taken in the same relative area between each septum. In total, thickness was measured at 24 points on each septum (Figure 1).

Each dissected septal cartilage was staged flat on a neutral background with a ruler as a fiducial marker. Photographs were taken perpendicular to the stage with a Canon EOS 70D (Canon, Tokyo, Japan) DSLR camera with a Zeiss Makro-Planar T 100 mm lens (Carl Zeiss, Oberkochen, Germany).

### 2.2. Shape Elucidation

**Figure 2:**
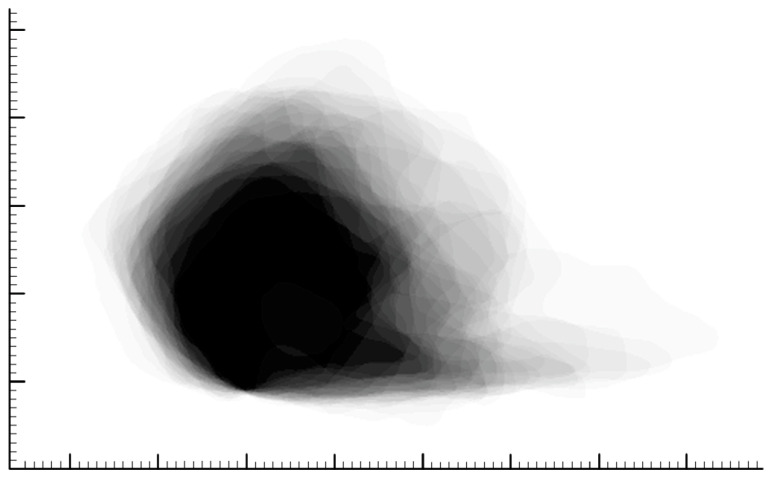
Overlay of all 40 cartilaginous septums after image processing in Photoshop. Black areas indicate that 100% of the septums occupy that space, while white areas indicate that 0% of the septums occupy that space. The average shape was determined by identifying the shape extracted when selecting for 50% gray. The small hash marks on the scale represent 1 mm.

**Figure 3:**
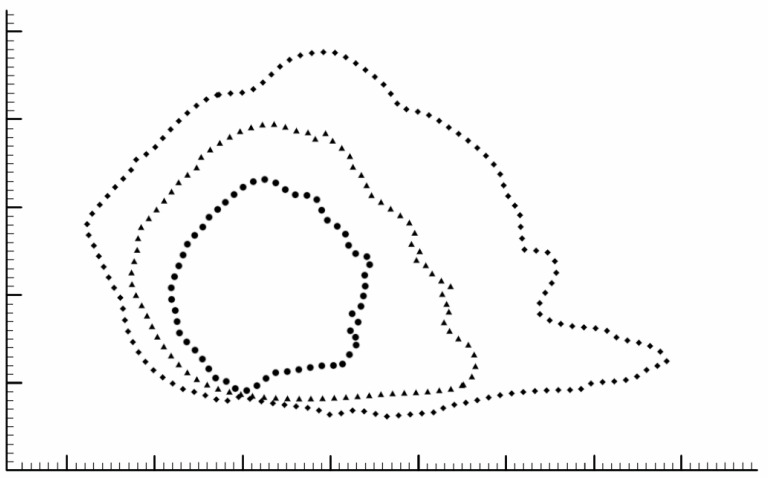
Mean cartilaginous septum shape, which was extracted from a composite image created by layering 40 cadaver septum images, is outlined by the triangles. The mean composite shape was extracted by selecting for the 50% gray image. The shapes defined by 95% and 5% of the septums were extracted in a similar fashion and are shown with circles and diamonds, respectively. The small hash marks on the scale represent 1 mm.

The pixel size was calibrated with the internal fiducial markers using Photoshop (CC 2018, Adobe, Mountain View, CA, USA). Image registration and anatomic co-registration were performed. The posterior caudal septal point (PCSP) of each cartilaginous septum was used for translational alignment, followed by rotational alignment with the posterior dorsal septal point (PDSP); this allowed for all the images to be aligned and rotated in the same way. Image processing was then performed, including the normalization of image sizes through Photoshop, the mapping of each septum, and a photograph pixel addition function to generate a histogram of the co-registered images. This essentially allowed us to overlay the silhouettes of all 40 septums, creating a composite shape. The overlay of all 40 septal silhouettes is depicted in a GIF file in the Supplementary Materials of this paper (Figure S1). The average shape, at the 50th percentile, was selected as the region where a mean of the septums overlapped; this corresponded to the shape extracted when selecting for 50% gray (Figure 2). In addition, variation in septal shape was plotted as the regions contained by 5% and 95% of the septums (Figure 3).

### 2.3. 3D Model Creation

The 50th-percentile cartilaginous septum shape was prepared for use as textures using Photoshop by overlaying the thickness measurements on the corresponding septum silhouette (Figure 1). Having all the measurement and shape images as layers in one file ensured a uniform scale of all the exported septum textures and served as a diagram for the textures and 3D model. The thickness measurements were converted into grayscale colors and textures were generated using Substance Designer (2019.1, Adobe, Mountain View, CA, USA), blending the values between the measurement points for a smooth result. Textures to visualize the thickness measurements as well as displacement maps for mesh generation were exported. Using Maya (2019.2, Autodesk, San Francisco, CA, USA), the septum shape and measurement texture were applied to planes and set to scale. The planes were cut to the septum shapes and extruded to the appropriate thickness using the displacement maps. The 3D septum model was sculpted between the measured points to create a smoother contour between known values. Secondary images of the septum were rendered with natural material and thickness visualizations.

## 3. Results

**Figure 4:**
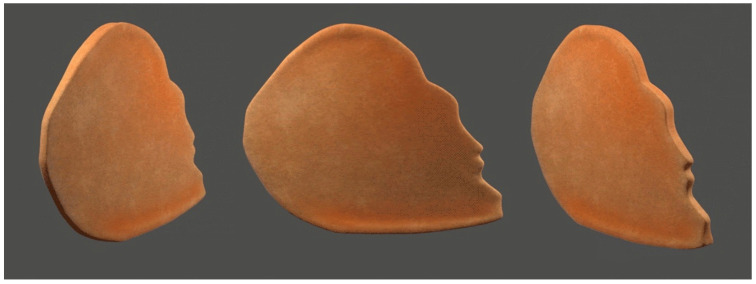
Different views of the mean 3D cartilaginous septum model based on the 50% shape and average thickness across 24 points. From left to right: anterior ¾ view, lateral view, and posterior ¾ view. The complete model can be accessed from the Supplementary Materials of this paper (S2) or at the NIH 3D model repository at the following link: https://3d.nih.gov/entries/3DPX-020598 (3DPX ID: 020598, Title: *3D Nasal Septum)*.

The primary result of this study is the mean 3D cartilaginous septum model, which can be found in the Supplemental Materials (Figure S2) or at the following link: https://3d.nih.gov/entries/3DPX-020598 (3DPX ID: 020598, Title: *3D Nasal Septum*). The model is presented as a mesh file in STL format. To create this model, 40 cartilaginous septums were used to determine the mean septal shape. The septal shapes for the 5th, 50th, and 95th percentiles were determined by adjusting the grayscale value (Figure 3). The length and height of the model based on the 50% shape (with the range between the 5% and 95% shapes) were 3.18 (1.84–6.55 cm) and 3.04 (2.04–4.24 cm), respectively. The average thickness of the 40 cadaveric septums ranged from 1.21 mm at the thinnest point in the center of the septum to 3.09 at the thickest point at the base of the septum (Figure 1). The 50% shape and the average thicknesses from all 24 points described in the methods were combined to develop the 3D model (Figure 4).

## 4. Discussion

### 4.1. Shape and Dimensions

In this study, we measured and photographed 40 cadaveric septums to develop a 3D model of the cartilaginous septum, accessible in the Supplementary Materials of this paper (S2) or at the NIH 3D model repository at the following link: https://3d.nih.gov/entries/3DPX-020598 (3DPX ID: 020598, Title: *3D Nasal Septum)*. To our knowledge, this study presents the largest anatomic septum cohort, a higher resolution of thickness than that seen in the literature, and the first evaluation of the mean shape of the cartilaginous septum (Figure 1). Apart from improving our general understanding of the septum, a quantified, mean septal shape allowed for the creation of an anatomically consistent 3D septum model. The average shape of our model accurately mimics the dimensions and proportions the cartilaginous septum, and it captures the diversity of the human septum better than any individual biological specimen. Unlike prior studies that presented a limited set of dimensional measurements, future researchers can take any measurements of the cartilaginous septum that they may need from our model that is publicly available at the NIH 3D repository.

There was wide variability in septum shape within our specimens, which is consistent with prior observations [6]. The variability in each individual septum is also observable in Supplementary Material S1, a GIF of the overlay of all 40 septums. Based on the shape elucidation when selecting for 5–95% gray, the shape changes drastically as the septum size changes (Figure 3). While the smaller 95% shape appears more “quadrangular”, the shape becomes more irregular as the size increases, with a “tail” of cartilage at the posterior-inferior edge of the septum increasing in length. While we only developed a 3D model based on the 50% shape in this study, the same methodology could be used to extrapolate a 3D model for each shape profile. This would allow future researchers and clinicians to select the appropriate model by estimating the septal size of their patients. Alternatively, the 50th percentile model could be resized in 3D software, although this may be an overgeneralization.

The dimensions of our model match those previously reported for the cartilaginous septum. Mowlavi et al. found the thickness of the cartilaginous septum from 11 cadavers to be thinnest at the center (mean ± SD = 1.3 ± 0.2 mm) and anterior septal angle (1.2 ± 0.1 mm), while the thickest point was at the base (2.7 ± 0.1 mm) [4]. Hwang et al. observed a similar pattern of thickness in their 14 cadaveric septums, with the thickness ranging from as a low as 0.74 mm at the center up to 3.03 mm at the base. They also reported an average length and height of 3.31 ± 0.53 and 2.99 ± 0.47 cm, respectively [2]. Similarly, our model has a length, height, and range of thickness of 3.18 cm, 3.04 cm, and 1.21–3.09 mm, respectively. The model accurately reflects the topography of the septum established by these prior studies: the thickest portions of the model followed bony-cartilaginous junctions and the septum transitioned from thick to thin to thick from anterior to posterior. Accurately modeling thickness is essential, as thickness is one of the most important determinants of septal strength [10]. The realistic dimensions of this model allow for numerous applications in future experimental design, clinical practice, and medical education.

### 4.2. Finite Elemental Analysis

The most immediate application of our cartilaginous septum model is to develop more consistent L-strut designs for use in finite elemental analyses (FEA). Determining the appropriate dimensions and shape of the L-strut is necessary for performing a successful septoplasty and several different designs exist. FEAs are often used to evaluate the biomechanics of digital L-strut designs—a force is applied and the stress throughout the structure is analyzed. Size, shape, and thickness are three of the primary inputs for an FEA and have been poorly studied in septal research. Our model, which is presented as a mesh file, represents an average of each of these inputs (Figure 4). Given that the L-strut is created from the leading edge of the cartilaginous septum, future studies can manipulate our mesh file to develop L-strut designs and test their strength using an FEA. Our model’s shape and thickness reflect the average dimensions of 40 cartilaginous septums. This offers several advantages over the previous septal models used in FEAs that were based on individual septums instead of composites.

While the L-strut is traditionally depicted and has been 3D modeled for use in FEAs as an angular “L”, the anatomic L-strut can more aptly be characterized as a “C”. The anterior edge of the septum has a known, curved rise from the traditionally straight dorsal and caudal struts [11,12,13]. While previous works have experimented with different L-strut designs, they often use the angular “L” shape as their basis. As a result, an FEA utilizing this artificial “L” shape may not capture dynamics that accurately translate to the human septum. A more recent paper partially rectified this discrepancy in shape by conducting an FEA on an L-strut model that mimicked the natural “C-curve”. This model not only better represented the anatomic septum but was also shown to better dissipate stress compared to traditional, angular L-strut designs. However, like other prior FEA studies on the cartilaginous septum, the models in this study were derived from the septal cartilage of just one cadaver [14]. Since septal shape shows significant variability between individuals, these studies may not be representative of the general patient population [6]. Furthermore, while studies have focused on the width of the dorsal and caudal struts, Liu et al. showed that thickness is a more important determinant of overall L-strut strength [15].

Our mean septal model accurately captures the “C” shape, reflects the diversity in shape better than any single septum, and has a fine resolution of thickness throughout the structure. Our 5–95% shapes can also be developed into 3D models in the future to provide even more accurate depictions of shape variability. This allows for a more accurate and generalizable representation of the septum and L-strut during experimentation. Thus, L-strut designs derived from our septum model will better reflect real-world dynamics, making FEA results more reproducible and applicable to clinical practice.

### 4.3. 3D Printing

The advent of 3D printing has allowed for improved trainee education and surgical outcomes and provides a myriad of possibilities within nasoseptal reconstruction [7]. In the following section, we review previous applications of 3DP to the nasal septum and identify ways in which our 3D model may advance these efforts.

Several training models have been developed with 3DP within the field of head and neck surgery, including temporal bone and larynx models which were used to replicate surgical dissections [7,9,16]. Similarly, AlReefi et al. developed a septoplasty training model, which was rated to be highly accurate by a cohort of rhinologists. However, this model was rendered utilizing the CT scan from only one patient [17]. Utilizing the mean septum in such a context would create an even more versatile training platform, as it would possess more detail and represent a wider patient population than a CT scan from a single patient.

Apart from education, 3DP in head and neck surgery has been used to produce custom reconstructive implants and, in the nose, is currently being investigated as a means for tissue engineering [7,9,18]. Nasal defects often require cartilage grafts harvested from the nasal septum, auricular cartilage, or costal cartilage. However, it is difficult to ensure these grafts are exactly the right shape, and larger donor site defects increase the risk of infection. Printing custom grafts would avoid creating a secondary surgical site and improve control over the shape. Recreating defects with 3DP often requires a standard model on which to base the structure. For example, patients with microtia have had prosthetics created by 3D-modeling and subsequently mirroring the unaffected ear [19,20,21]. However, since the septum has no anatomic counterpart that can be mirrored, patient-specific imaging in conjunction with a mean septal model would allow for anatomically consistent grafts. 

These reconstructions may then be bioprinted, a process that allows for the deposition of live cells directly to a desired location, allowing for tissue regeneration [22]. Cao et al. explain that bioprinting requires design of a scaffold, which is usually based on patient images. Once the scaffold is 3D-printed, cells can be cultured onto it in vitro and subsequently implanted into the patient. Several artificial and natural materials may be used as scaffolds and show potential in their ability to reconstruct nasal cartilage [8].

One such material is polycaprolactone (PCL). This bioresorbable polymer is porous and can be seeded with cells that eventually replace the scaffold with native-appearing tissue. Zopf et al. utilized 3DP to create PCL nasal scaffolds, which, when implanted into a porcine model subcutaneously, maintained an appropriate shape and showed complete soft tissue ingrowth [23]. PCL has also been used during rhinoplasty as a septal extension graft [24]. In septoplasties, this material offers an alternative to traditional autologous graphs, which weaken the inherent strength of the septum and lead to nasal dorsum collapse over time. 3DP enables precise control over the nanostructure of PCL, ensuring nasal implants are strong enough to fix the septal defect, yet not too stiff to avoid nasal tip necrosis, and still thin enough to avoid narrowing the internal nasal valve. Kim et al. used 3D-printed PCL septal implants with the appropriate porosity to repair caudal septal deviations. As the grafts degraded, scar tissue formed and prevented deformation. These implants showed short- and long-term success in repairing septal deviations [25,26]. Combining the technique and materials described in Kim et al. with a mean 3D septum model would allow not only for control over the nanostructure of the septum, but also the overall shape of the macrostructure, potentially improving the fit of these implants.

Another reconstructive application of 3DP is the repair of nasal septal perforations. Currently, septal perforations are typically repaired with an implanted graft, often composed of autologous cartilage (i.e., auricular cartilage) or synthetic material, sandwiched between bilateral mucoperichondrial flaps [27]. Given the limited vascular supply in the area, grafts greater than 2 cm often have low success rates requiring larger reconstructions using local or free flaps. Septal buttons–silicone coverings–can also be used for large perforations, but these are often associated with discomfort and crusting, often leading to poor patient adherence [28]. 

3D modeling has expanded the repertoire available for treatment of these defects. Onerci Altunay et al. used patient imaging to create 3D-printed silastic septal buttons that closely matched the size and shape of their patients’ septal perforations. These fitted prosthetics had a 90% retention rate, exceeding the rates prior to 3D-sizing [29]. Gadaleta et al. made the first step towards tissue engineering cartilage grafts for septal perforations by similarly using CT images to design a PCL scaffold that matched the size and shape of the defect. The PCL scaffold thickness was based on the average thickness of the septum and maintained constant throughout the structure. The scaffolds were not implanted, but they could theoretically be seeded with cells to introduce cartilage growth at the site of the defect [27]. These surgeons were able to recreate the sagittal shape of the defect based on patient imaging. However, the thickness of the septum, which, as mentioned earlier, is variable throughout, was not captured in these implants. Using a graft that better mimics the actual thickness of the septum at any given location may ensure a more appropriate match between the vascular demands of the graft and the vascular supply at that point on the septum. Thus, a graft based on our standardized model of the cartilaginous septum, which captures average thickness across several points, in conjunction with patient imaging, may maximize the chances of graft survival.

### 4.4. Limitations and Future Directions

Our mean shape analysis was limited by the number of septums (*n* = 40) that were dissected. A future, more comprehensive shape analysis could be higher powered by a larger number of dissections or the use of technology to accurately measure the septum in three dimensions. Further studies could also measure the thickness of the septum at more points (in this study, *n* = 24 points per septum) to capture the contour of the shape in finer detail. Another focus of future studies could be on the variability in shape within different sized subgroups, as variability among larger septums may differ from that of smaller septums. Finally, while our model captures the average dimensions of the septum, the chemical composition of the septum also varies throughout [30]. Future studies overlaying this data onto our average septum could create an even more accurate model.

## 5. Conclusions

In this study, we devised a method to show the average shape of the cartilaginous nasal septum from 40 cadaveric septums. To the best of our knowledge, this is the first attempt to capture the average shape of this highly variable anatomical structure. We then combined the average shape with thickness measurements to develop a 3D model. Since this model is an average of multiple septums, it establishes an anatomical standard for the cartilaginous septum. Researchers can utilize this model to conduct fininite elemental analyses to generate reproducible, generalizable results. Furthermore, past works have shown that 3D printing has great potential within the field of otolaryngology. The 3D printing of our model may advance these efforts by approximating natural anatomy and aesthetics, thus providing an accurate scaffold for educational models, implantable prosthetics, and cartilage regeneration.

## Data Availability

The 3D model is found in the Supplementary Materials and will be uploaded to NIH 3D website with a link provided.

## Supplementary Materials

The following supporting information can be downloaded at: https://www.mdpi.com/article/10.3390/biomimetics8070530/s1, Figure S1: GIF of septum overlay; Figure S2: STL file of 3D cartilaginous nasal septum model.
