# Supplementary figures and images for: The Creation of an Average 3D Model of the Human Cartilaginous Nasal Septum and Its Biomimetic Applications
Source: Biomimetics (Basel). 2023 Nov 6;8(7):530. doi: 10.3390/biomimetics8070530 (PMC10669719; doi:10.3390/biomimetics8070530)

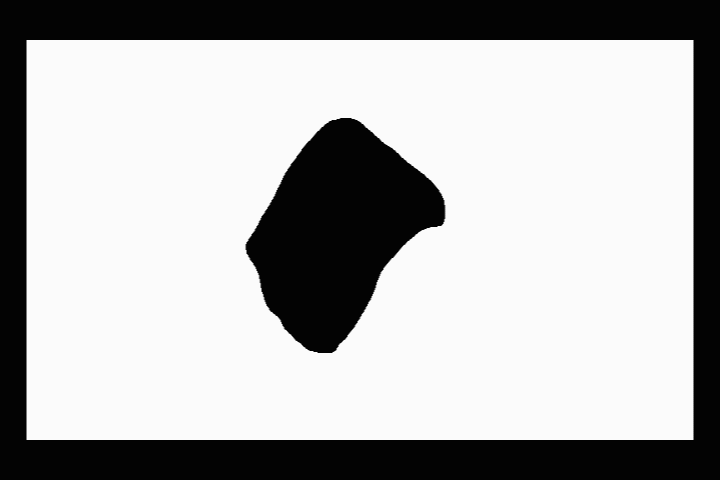

Supplement: Supplementary file 1 [file biomimetics-08-00530-s001.zip › S1.gif]
